# *Vital Signs:* Postpartum Depressive Symptoms and Provider Discussions About Perinatal Depression — United States, 2018

**DOI:** 10.15585/mmwr.mm6919a2

**Published:** 2020-05-15

**Authors:** Brenda L. Bauman, Jean Y. Ko, Shanna Cox, Denise V. D’Angelo, MPH, Lee Warner, Suzanne Folger, Heather D. Tevendale, Kelsey C. Coy, Leslie Harrison, Wanda D. Barfield

**Affiliations:** 1 Division of Reproductive Health, National Center for Chronic Disease Prevention and Health Promotion, CDC.

## Abstract

**Introduction:**

Perinatal depression is a complication of pregnancy that can result in adverse maternal and infant outcomes. Screening to identify pregnant and postpartum women with depressive symptoms is recommended to provide diagnosis, treatment, and follow-up care to reduce poor outcomes.

**Methods:**

CDC analyzed 2018 data from the Pregnancy Risk Assessment Monitoring System to describe postpartum depressive symptoms (PDS) among women with a recent live birth and to assess whether health care providers asked women about depression during prenatal and postpartum health care visits, by site and maternal and infant characteristics.

**Results:**

Among respondents from 31 sites, the prevalence of PDS was 13.2%, ranging from 9.7% in Illinois to 23.5% in Mississippi. The prevalence of PDS exceeded 20% among women who were aged ≤19 years, were American Indian/Alaska Native, smoked during or after pregnancy, experienced intimate partner violence before or during pregnancy, self-reported depression before or during pregnancy, or whose infant had died since birth. The prevalence of women reporting that a health care provider asked about depression during prenatal care visits was 79.1% overall, ranging from 51.3% in Puerto Rico to 90.7% in Alaska. The prevalence of women reporting that a provider asked about depression during postpartum visits was 87.4% overall, ranging from 50.7% in Puerto Rico to 96.2% in Vermont.

**Conclusions and Implications for Public Health Practice:**

The prevalence of self-reported PDS varied by site and maternal and infant characteristics. Whether providers asked women about perinatal depression was not consistent across sites. Provision of recommended screenings and appropriate referrals for diagnosis, treatment, and follow-up care can ensure early and effective management of depression to reduce adverse maternal and infant outcomes.

## Introduction

Mental health conditions are common complications in pregnancy (*1*) and an underlying cause for approximately 9% of pregnancy-related deaths (*2*). Postpartum depression is associated with lower rates of breastfeeding initiation, poorer maternal and infant bonding, and increased likelihood of infants showing developmental delays (*3*). Left untreated, postpartum depression can adversely affect the mother’s health and might cause sleeping, eating, and behavioral problems for the infant; when effectively treated and managed, both mother and child benefit (*4*).

Professional and clinical organizations have issued recommendations to address perinatal (i.e., during and after pregnancy) depression. The United States Preventive Services Task Force (USPSTF) recommends that all adults be screened for depression, including pregnant and postpartum women (*5*), and that clinicians provide or refer pregnant and postpartum women who are at increased risk for perinatal depression to counseling interventions (*6*). The American College of Obstetricians and Gynecologists (ACOG) recommends that obstetric care providers screen patients for depression and anxiety symptoms at least once during the perinatal period and also conduct a full assessment of mood and emotional well-being during the comprehensive postpartum visit (*7*). If a patient is screened for depression and anxiety during pregnancy, additional screening should also occur during the comprehensive postpartum visit (*7*). The American Academy of Pediatrics also recommends that routine screening for maternal postpartum depression be integrated into well-child visits (*8*).

USPSTF has noted that identifying women at increased risk for perinatal depression and determining ways to improve the delivery of interventions represent evidence gaps that warrant high-priority efforts (*9*). Women with postpartum depressive symptoms (PDS) are at increased risk for postpartum depression and require further evaluation to determine whether they meet the criteria for having a depressive disorder (*4*). To inform these evidence gaps, CDC used data from the Pregnancy Risk Assessment Monitoring System (PRAMS) to examine the prevalence of self-reported PDS and whether a health care provider inquired about depression during prenatal and postpartum health care visits.

## Methods

PRAMS collects site-specific, population-based data on self-reported maternal behaviors and experiences before, during, and shortly after pregnancy. From each of the 50 continuously participating sites, a stratified, random sample of women with a recent live birth (singleton and multiple births) is selected monthly from birth certificate files, and these women are surveyed 2–6 months postpartum (average = 4 months) using a standardized protocol and questionnaire (*10*). Annually, PRAMS data for each site are weighted for sampling design, nonresponse, and noncoverage to produce data representative of the site’s birth population for the year.

Data from 31 PRAMS sites* that had weighted response rates ≥55% in 2018 were included in this analysis. Data were obtained from the infant’s birth certificate and survey questions.^†^ Self-reported PDS were ascertained by categorizing five responses (“always,” “often,” “sometimes,” “rarely,” and “never”) from the following two questions adapted from the Patient Health Questionnaire-2 screening instrument (*11*): 1) “Since your new baby was born, how often have you felt down, depressed, or hopeless?” and 2) “Since your new baby was born, how often have you had little interest or little pleasure in doing things?” Women responding “always” or “often” to either question were classified as experiencing PDS. Women who had prenatal and postpartum health care visits were asked whether health care providers had inquired about depression during these visits. Health care provider inquiry about depression during prenatal care visits was ascertained by the percentage of women responding “yes” to the question “During any of your prenatal care visits, did a doctor, nurse, or health care worker ask you if you were feeling down or depressed.” Health care provider inquiry about depression during postpartum visits was assessed by the percentage of women responding “yes” to the question, “During your postpartum checkup, did a doctor, nurse, or other health care worker ask if you were feeling down or depressed.”

The weighted prevalence and 95% confidence intervals for self-reported PDS and health care provider inquiry about depression during prenatal and postpartum visits were calculated overall and by site. Chi-squared tests of independence were used to examine the distribution of both PDS and health care provider inquiry about depression by selected maternal characteristics (age, race/ethnicity, education level, marital status, participation in the Special Supplemental Nutrition Program for Women, Infants, and Children [WIC] during pregnancy, health insurance at delivery,^§^ and number of previous live births) and behaviors and experiences (smoking status during the last 3 months of pregnancy or the postpartum period, experience of intimate partner violence before or during pregnancy, and self-reported depression before or during pregnancy). Chi-squared tests of independence were examined for PDS only for selected maternal characteristics (breastfeeding initiation and duration and having a health care provider ask about depression during prenatal and postpartum visits) and infant characteristics (infant’s gestational age at birth and infant vital status at survey completion). Subgroup differences in PDS and health care provider inquiry about depression during prenatal and postpartum visits were ascertained using 95% confidence interval^¶^ estimates of the weighted prevalence.

CDC tested for linear trends in aggregate estimates of PDS from 2012 to 2018 among 16 continuously reporting sites** and linear trends in health care providers asking about depression during prenatal and postpartum visits from 2016 to 2018 for 22 continuously reporting sites,^††^ using logistic regression, adjusting for site. All statistical analyses were conducted using a SAS (version 9.4; SAS Institute) complex survey module to account for the PRAMS sampling design.

## Results

Among respondents from 31 PRAMS sites, the prevalence of self-reported PDS was 13.2%, ranging from 9.7% (Illinois) and 10.3% (Massachusetts) to 19.4% (West Virginia) and 23.5% (Mississippi) (Table 1). Among 16 continuously reporting sites, a small but statistically significant annual percentage point increase of 0.22% (p-value <0.05) in PDS was observed from 2012 to 2018. PDS prevalence varied by selected demographic and other maternal characteristics (Table 2). Prevalence was higher among women aged ≤19 and 20–24 years, those who were non-Hispanic black (black), non-Hispanic American Indian/Alaska Native (American Indian/Alaska Native) or non-Hispanic Asian/Pacific Islander (Asian/Pacific Islander), who had completed ≤12 years of education, and were not married (includes living with partner), than among those aged 25–34 and ≥35 years, who were non-Hispanic white (white) or Hispanic, had completed >12 years of education and were married. Prevalence was also higher among women who had participated in WIC during pregnancy, had Medicaid at delivery, smoked cigarettes during the last 3 months of pregnancy or postpartum, breastfed their infants for <8 weeks, had experienced intimate partner violence before or during pregnancy, self-reported depression before or during pregnancy, or whose infant had died since birth, compared with women who had not participated in WIC, had private health insurance, had not smoked during the last trimester or postpartum, breastfed their infants for ≥8 weeks, had not experienced intimate partner violence, had not experienced depression before or during pregnancy, and whose infant was alive at the time of the survey. PDS prevalence exceeded 20% among women aged ≤19 years, American Indians/Alaska Natives, women who smoked during or after pregnancy, experienced intimate partner violence or depression before or during pregnancy, or whose infant had died since birth.

**TABLE 1:** Prevalence of self-reported postpartum depressive symptoms among women with a recent live birth, 31 sites – Pregnancy Risk Assessment Monitoring System (PRAMS), 2018

Site	No.*	Postpartum depressive symptoms %^†^ (95% CI)
**All 31 sites**	**32,659**	**13.2 (12.6–13.8)**
Alaska	974	14.8 (12.2–17.3)
Colorado	1,117	11.1 (8.8–13.3)
Connecticut	1,380	11.7 (9.6–13.7)
Delaware	824	13.1 (10.7–15.5)
Georgia	752	13.6 (10.4–16.7)
Illinois	1,298	9.7 (7.9–11.4)
Kansas	958	14.7 (11.6–17.7)
Kentucky	738	14.0 (10.7–17.2)
Louisiana	844	15.9 (13.3–18.6)
Maine	812	10.9 (8.2–13.5)
Massachusetts	1,412	10.3 (8.4–12.2)
Michigan	1,790	16.4 (14.2–18.5)
Minnesota	1,262	10.6 (8.5–12.7)
Mississippi	1,169	23.5 (20.5–26.6)
Missouri	921	13.7 (11.2–16.3)
Nebraska	1,293	12.1 (9.5–14.7)
New Jersey	1,151	11.2 (9.3–13.0)
New Mexico	1,194	15.3 (13.2–17.4)
New York City	1,469	15.5 (13.2–17.7)
North Dakota	865	11.7 (9.1–14.3)
Pennsylvania	934	14.7 (12.0–17.4)
Puerto Rico	943	10.8 (8.3–13.3)
Rhode Island	1,061	12.3 (9.9–14.6)
South Dakota	995	13.0 (10.7–15.3)
Utah	1,222	14.7 (12.3–17.0)
Vermont	848	10.7 (8.5–12.9)
Virginia	1,126	13.5 (10.1–16.8)
Washington	1,100	11.4 (9.2–13.5)
West Virginia	681	19.4 (15.9–22.9)
Wisconsin	988	10.5 (8.0–12.9)
Wyoming	538	15.7 (12.0–19.5)

**Abbreviation:** CI = confidence interval.

* Unweighted sample size.

^†^ Weighted percentage.

**TABLE 2:** Prevalence of self-reported postpartum depressive symptoms among women with a recent live birth, by selected characteristics — Pregnancy Risk Assessment Monitoring System (PRAMS), 31 sites,* 2018

Characteristic	Postpartum depressive symptoms (N = 32,659)^†^ %^§^ (95% CI)
**Age group (yrs)^¶^**	
≤19	22.2 (18.8–25.6)
20–24	17.8 (16.3–19.4)
25–34	11.9 (11.2–12.6)
≥35	10.8 (9.7–12.0)
**Race/Ethnicity^¶^**	
White, non-Hispanic	11.4 (10.7–12.1)
Black, non-Hispanic	18.2 (16.5–19.9)
Hispanic	12.0 (10.8–13.2)
American Indian/Alaska Native, non-Hispanic	22.0 (17.7–26.3)
Asian/Pacific Islander, non-Hispanic	19.2 (16.6–21.7)
Other, non-Hispanic	16.3 (13.1–19.5)
**Education level (yrs)^¶^**	
<12	17.8 (15.8–19.7)
12	16.2 (14.9–17.5)
>12	11.2 (10.6–11.9)
**Marital status^¶^**	
Married	11.0 (10.3–11.6)
Not married**	16.9 (15.9–17.9)
**WIC participation during pregnancy^¶^**	
Yes	17.0 (15.9–18.0)
No	11.2 (10.6–11.9)
**Health insurance at delivery^¶^**	
Private	10.1 (9.5–10.8)
Medicaid	17.2 (16.3–18.2)
None	13.2 (10.0–16.3)
**No. of previous live births**	
First birth	13.2 (12.3–14.1)
Second or later birth	13.2 (12.5–13.9)
**Smoked cigarettes during last 3 mos of pregnancy^¶^**	
Yes	22.3 (19.7–24.8)
No	12.4 (11.9–13.0)
**Smoked cigarettes postpartum^¶^**	
Yes	21.5 (19.4–23.6)
No	12.2 (11.6–12.8)
**Any intimate partner violence before/during pregnancy^¶,††^**	
Yes	33.1 (28.7–37.4)
No	12.5 (11.9–13.0)
**Breastfeeding duration^¶^**	
Breastfed ≥8 wks	11.8 (11.1–12.4)
Breastfed <8 wks	15.6 (14.2–17.0)
Never breastfed	14.0 (12.3–15.6)
**Infant gestational age at birth (wks)^¶^**	
Preterm (<37)	17.1 (15.5–18.8)
Term (≥37)	12.8 (12.2–13.4)
**Infant vital status at survey completion^¶^**	
Alive	13.0 (12.4–13.5)
Deceased	48.7 (39.3–58.1)
**Self-reported depression before pregnancy^¶^**	
Yes	28.7 (26.7–30.7)
No	10.6 (10.1–11.2)
**Self-reported depression during pregnancy^¶^**	
Yes	34.3 (32.2–36.5)
No	9.9 (9.4–10.5)
**HCP asked about depression during prenatal visit^¶^**	
Yes	12.7 (12.0–13.3)
No	14.5 (13.2–15.8)
**HCP asked about depression during postpartum visit^¶^**	
Yes	12.3 (11.6–12.9)
No	14.3 (12.5–16.0)

**Abbreviations:** CI = confidence interval; HCP = health care provider; WIC = Special Supplemental Nutrition Program for Women, Infants, and Children.

* Alaska, Colorado, Connecticut, Delaware, Georgia, Illinois, Kansas, Kentucky, Louisiana, Maine, Massachusetts, Michigan, Minnesota, Mississippi, Missouri, Nebraska, New Jersey, New Mexico, New York City, North Dakota, Pennsylvania, Puerto Rico, Rhode Island, South Dakota, Utah, Vermont, Virginia, Washington, West Virginia, Wisconsin, and Wyoming.

^†^ Unweighted sample size.

^§^ Weighted percentage.

^¶^ p<0.05 from chi-squared test of independence.

** Includes single status or living with partner.

^††^ Includes intimate partner violence from current husband/partner or ex-husband/ex-partner.

Nearly all women (99.2%) received prenatal care; 79.1% of those who received prenatal care reported being asked by a health care provider about depression during pregnancy. The prevalence of health care provider inquiry about depression during prenatal visits varied by site, ranging from 51.3% (Puerto Rico) and 69.4% (Mississippi) to 90.6% (Minnesota) and 90.7% (Alaska) (Table 3).

**TABLE 3:** Prevalence of health care providers asking about depression during prenatal and postpartum visits as reported by women with a recent live birth — Pregnancy Risk Assessment Monitoring System (PRAMS), 31 sites, 2018

Site	Health care providers asked about depression %* (95% CI)	
	Prenatal visit (n = 32,619)^†^	Postpartum visit (n = 29,187)^†^
**All 31 sites**	**79.1 (78.4–79.7)**	**87.4 (86.9–88.0)**
Alaska	90.7 (88.5–92.8)	94.2 (92.4–96.0)
Colorado	82.5 (79.9–85.0)	93.0 (91.2–94.8)
Connecticut	73.5 (70.3–76.6)	89.8 (87.6–92.1)
Delaware	85.1 (82.5–87.6)	88.7 (86.2–91.1)
Georgia	79.1 (75.3–82.9)	85.8 (82.3–89.2)
Illinois	82.2 (79.9–84.5)	91.7 (90.0–93.4)
Kansas	77.9 (74.6–81.2)	85.2 (82.3–88.2)
Kentucky	69.7 (65.3–74.1)	85.4 (81.8–88.9)
Louisiana	70.8 (67.4–74.2)	75.0 (71.7–78.4)
Maine	90.5 (88.1–92.8)	95.5 (93.8–97.3)
Massachusetts	82.7 (80.1–85.3)	93.6 (91.9–95.2)
Michigan	83.4 (81.2–85.6)	88.6 (86.6–90.5)
Minnesota	90.6 (88.7–92.5)	95.9 (94.5–97.3)
Mississippi	69.4 (66.1–72.7)	76.9 (73.8–80.1)
Missouri	77.9 (74.9–81.0)	85.2 (82.4–88.0)
Nebraska	86.3 (83.6–89.0)	89.8 (87.4–92.3)
New Jersey	71.6 (68.8–74.4)	84.8 (82.5–87.1)
New Mexico	89.1 (87.3–90.9)	93.7 (92.2–95.2)
New York City	71.2 (68.4–73.9)	73.1 (70.2–76.0)
North Dakota	89.6 (87.2–92.0)	94.1 (92.1–96.2)
Pennsylvania	81.4 (78.6–84.2)	90.7 (88.4–93.0)
Puerto Rico	51.3 (47.3–55.3)	50.7 (46.1–55.2)
Rhode Island	83.9 (81.4–86.4)	91.8 (89.9–93.7)
South Dakota	87.1 (84.6–89.6)	95.0 (93.4–96.7)
Utah	69.5 (66.3–72.8)	87.3 (84.8–89.7)
Vermont	89.6 (87.6–91.7)	96.2 (94.9–97.6)
Virginia	77.0 (73.0–81.1)	90.3 (87.2–93.3)
Washington	84.8 (82.2–87.4)	91.1 (89.1–93.2)
West Virginia	78.6 (74.8–82.3)	82.4 (78.7–86.1)
Wisconsin	85.8 (82.8–88.9)	90.9 (88.2–93.6)
Wyoming	80.2 (76.1–84.3)	85.9 (82.2–89.7)

**Abbreviation:** CI = confidence interval.

* Weighted percentage.

^†^ Unweighted sample size

The percentage of women who reported that a health care provider asked about depression during prenatal visits was higher among respondents aged ≤19 and 20–24 years than among those aged ≥25 years, and was higher among those who were black, Hispanic, American Indian/Alaska Native, or non-Hispanic other (other) than among respondents who were white or Asian/Pacific Islander. Prevalence was also higher among those who had ≤12 years of education, were not married, had participated in WIC, had Medicaid at delivery, smoked cigarettes during the last trimester of pregnancy, or self-reported depression before or during pregnancy compared with those who had >12 years of education, were married, had not participated in WIC, had private or no health insurance, had not smoked during the last trimester of pregnancy, or had not experienced depression before or during pregnancy (Table 4). Among 22 continuously reporting sites, the prevalence of health care provider inquiry about depression during prenatal visits increased significantly during 2016–2018, from 76.2% to 79.3% (p <0.05), with an average annual percentage point increase of 1.5% (data not shown).

**TABLE 4:** Prevalence of health care providers asking about depression during prenatal and postpartum visits as reported by women with a recent live birth, by selected characteristics — Pregnancy Risk Assessment Monitoring System (PRAMS), 31 sites,* 2018

Characteristic	Health care providers asked about depression %^†^ (95% CI)	
	Prenatal visits (n = 32,619)^§^	Postpartum visit (n = 29,187)^§^
Age group (yrs)^¶,^**		
≤19	86.9 (84.0–89.7)	91.3 (89.4–93.3)
20–24	83.2 (81.7–84.7)	87.8 (86.4–89.2)
25–34	78.6 (77.7–79.5)	87.4 (86.7–88.2)
≥35	74.9 (73.3–76.6)	86.4 (85.1–87.7)
Race/Ethnicity^¶,^**		
White, non-Hispanic	76.7 (75.8–77.6)	88.1 (87.3–88.8)
Black, non-Hispanic	85.5 (84.0–87.0)	86.8 (85.2–88.4)
Hispanic	81.8 (80.4–83.2)	86.2 (84.8–87.5)
American Indian/Alaska Native, non-Hispanic	91.5 (88.2–94.8)	92.2 (88.3–96.1)
Asian/Pacific Islander, non-Hispanic	74.6 (71.8–77.5)	83.0 (80.6–85.5)
Other, non-Hispanic	82.8 (79.6–86.1)	91.4 (88.5–94.3)
**Education level (yrs)^¶^**		
<12	84.4 (82.6–86.2)	87.7 (85.9–89.6)
12	83.2 (81.9–84.6)	87.1 (85.8–88.4)
>12	76.5 (75.7–77.4)	87.4 (86.8–88.1)
**Marital status^¶^**		
Married	75.8 (74.9–76.7)	87.2 (86.5–87.9)
Not married^††^	84.5 (83.5–85.5)	87.9 (86.9–88.8)
**WIC participation during pregnancy^¶^**		
Yes	84.1 (83.0–85.1)	88.2 (87.3–89.1)
No	76.4 (75.6–77.3)	87.1 (86.4–87.8)
**Health insurance at delivery^¶^**		
Private	75.2 (74.3–76.2)	87.2 (86.5–88.0)
Medicaid	84.3 (83.3–85.2)	87.9 (87.0–88.8)
None	77.3 (73.2–81.4)	86.9 (83.4–90.4)
No. of previous live births**		
First birth	78.4 (77.3–79.5)	88.4 (87.5–89.2)
Second or later birth	79.5 (78.7–80.3)	86.9 (86.2–87.6)
**Smoked cigarettes during last 3 mos of pregnancy^¶^**		
Yes	83.7 (81.4–86.1)	87.7 (85.2–90.1)
No	78.7 (78.0–79.4)	87.5 (86.9–88.0)
**Smoked cigarettes postpartum**		
Yes	N/A	87.9 (85.9–89.9)
No		87.5 (86.9–88.1)
**Any intimate partner violence before/during pregnancy^§§^**		
Yes	80.5 (76.9–84.2)	85.9 (82.4–89.3)
No	79.0 (78.3–79.7)	87.6 (87.0–88.1)
Self-reported depression before pregnancy^¶,^**		
Yes	86.2 (84.7–87.7)	90.5 (89.0–91.9)
No	77.9 (77.2–78.7)	87.0 (86.4–87.7)
Self-reported depression during pregnancy^¶,^**		
Yes	85.5 (83.9–87.1)	90.7 (89.3–92.2)
No	78.1 (77.4–78.8)	87.0 (86.4–87.6)

**Abbreviations:** CI = confidence interval; N/A = not applicable; WIC = Special Supplemental Nutrition Program for Women, Infants, and Children.

* Alaska, Colorado, Connecticut, Delaware, Georgia, Illinois, Kansas, Kentucky, Louisiana, Maine, Massachusetts, Michigan, Minnesota, Mississippi, Missouri, Nebraska, New Jersey, New Mexico, New York City, North Dakota, Pennsylvania, Puerto Rico, Rhode Island, South Dakota, Utah, Vermont, Virginia, Washington, West Virginia, Wisconsin, and Wyoming.

^†^ Weighted percentage.

^§^ Unweighted sample size.

^¶^ p<0.05 from chi-squared test of independence for the prenatal period.

** p<0.05 from chi-squared test of independence for the postpartum period.

^††^ Includes single status or living with partner.

^§§^ Includes intimate partner violence from current husband/partner or ex-husband/ex-partner.

Overall, 90.1% of women attended a postpartum visit, among whom 87.4% reported being asked by a health care provider about depression during the visit. The percentage of women reporting that their health care provider asked about depression during a postpartum visit varied by site, ranging from 50.7% (Puerto Rico) and 73.1% (New York City) to 95.9% (Minnesota) and 96.2% (Vermont) (Table 3). The reported percentage of having a health care provider ask about depression during a postpartum visit was higher among women aged ≤19 years (compared with women aged 20–24, 25–34 or ≥35 years), who were white, American Indian/Alaska Native, or other (compared with those who were Asian/Pacific Islander), and among those who self-reported depression before or during pregnancy (Table 4). Among 22 continuously reporting sites, the prevalence of health care provider inquiry about depression during the postpartum visit increased significantly from 84.1% to 88.0% (p<0.05) during 2016–2018, with an average annual percentage point increase of 1.8% (data not shown).

## Discussion

In this survey of women with a recent live birth from 31 PRAMS sites, approximately one in eight reported experiencing postpartum depressive symptoms since their infant’s birth; PRAMS responses are reported an average of 4 months postpartum, which suggests persistence of these symptoms. The observed variation in PDS by PRAMS sites and selected characteristics is similar to that found in previous reports using PRAMS data (*12*). Differences in the prevalence of PDS by site might reflect differences in the distribution of risk factors, such as low socioeconomic status (*13*). In some subgroups, approximately 20% of women reported PDS, including those aged ≤19 years, who were American Indian/Alaska Native, smoked cigarettes during pregnancy or postpartum, experienced intimate partner violence before or during pregnancy, or self-reported depression before or during pregnancy.

Women with postpartum depression are more likely to have a diagnosis of depression either before or during pregnancy (*14*). In this analysis, the percentage of women with PDS was similarly higher among those who self-reported depression before or during pregnancy; this might reflect the continuum of the condition across the preconception and perinatal period. This study used an adaption of two items from the Patient Health Questionnaire-2, an evidence-based tool used to screen for current depressive symptoms. If the criteria for positive symptomology are met using this tool in clinical practice, the patient should receive further assessment to determine whether a diagnosis of major depressive episode is warranted (*4*). Identifying women with PDS should be complemented with adequate systems to ensure needed diagnosis, treatment, and follow-up (*5*). One study from the National Survey of Drug Use and Health indicated that past-year major depressive episodes are common in both pregnant (7.7%) and nonpregnant (11.1%) females of reproductive age (*15*). Furthermore, regardless of pregnancy status, as many as 60% of these persons did not receive a clinical diagnosis and only one half received treatment (*15*). To optimize the health of women and infants, postpartum care should become an ongoing process, with services and support tailored to each woman’s individual needs. The comprehensive postpartum visit should include a full assessment of physical, social, and psychological well-being. Women with chronic medical conditions should be counseled regarding the importance of timely follow-up with their obstetrician–gynecologist or primary care provider for ongoing coordination of care (*16*).

The prevalence of inquiry about depression by a health care provider was higher during postpartum than prenatal visits, both overall and in 21 (68%) of 31 participating sites. The emphasis in ACOG recommendations for a full assessment of mood and emotional well-being in the postpartum period (*7*) and less evidence of the benefit of screening pregnant versus postpartum women for depression (*5*) might explain some of these differences. Although universal screening for depression is recommended for pregnant and postpartum women (*6*,*7*), variation was seen in the percentage of women who reported being asked about depression by the characteristics assessed. Despite the observed increase in the percentage of health care providers asking women about depression over time, one in eight women with a live birth in 2018 reported not being asked about depression during a postpartum visit, and one in five did not report being asked at a prenatal visit. Health care providers can provide timely perinatal depression education to women and family members or other support persons.^§§^ Health systems can implement quality improvement through screening and linkage to care for depression during both the prenatal and postpartum periods (*17*).

Variation in site-based estimates of the percentage of health care providers who asked about depression might be related to differences in state initiatives to increase provider capacity and link women to care. For example, state-based programs such as the Massachusetts Child Psychiatry Access Program for Moms aim to increase the capacity of obstetric providers to address perinatal depression in health care settings (*18*). The Health Resources and Services Administration recently funded seven states to implement programs to support providers, through real-time psychiatric consultation, care coordination, and training in screening, assessing, referring, and treating pregnant and postpartum women for depression and other behavioral health conditions.^¶¶^ Additional state-level programmatic initiatives can be leveraged to address perinatal depression through programs such as Healthy Start,*** home visiting,^†††^ and Title V.^§§§^

The findings in this report are subject to at least five limitations. First, results are only representative of women with a recent live birth in the PRAMS sites that are included in the report. Second, postpartum depressive symptoms were self-reported and are not necessarily indicative of a clinical diagnosis of depression. Third, estimates might not capture depressive symptoms that resolved before or began after survey completion, which occurred an average of approximately 4 months after the live birth. Fourth, self-reported PDS and provider discussions about depression are subject to both recall and social desirability biases. Finally, the study assessed health care provider inquiry about depression during prenatal and postpartum visits, but these data cannot provide knowledge of whether recommended screening and referrals were performed or of the content of any care provided outside of the health care setting.

Perinatal depression is a common complication of pregnancy that can be addressed at multiple levels. Screening for perinatal depression should be accompanied by evidence-based systems for diagnosis, counseling, treatment, and referral. Ongoing, site-specific surveillance with PRAMS can be used to monitor estimates of PDS and provider discussions about depression in the perinatal period and identify opportunities for providers, health systems, and states to better support women and their families.

**Summary**

**What is already known about this topic?**

Perinatal depression is a complication of pregnancy associated with poor maternal and infant health outcomes. Universal screening of pregnant and postpartum women for depression is recommended.

**What is added by this report?**

Although 13% of surveyed women with a recent live birth reported depressive symptoms during the postpartum period, one in five did not report a health care provider asking about depression during prenatal visits and one in eight reported they were not asked about depression during postpartum visits.

**What are the implications for public health practice?**

Health care provider screening of all women in the perinatal period can increase identification of women at risk for depression and provision of care or referral for appropriate diagnosis and treatment.

## Footnotes

* The 31 sites include Alaska, Colorado, Connecticut, Delaware, Georgia, Illinois, Kansas, Kentucky, Louisiana, Maine, Massachusetts, Michigan, Minnesota, Mississippi, Missouri, Nebraska, New Jersey, New Mexico, New York City, North Dakota, Pennsylvania, Puerto Rico, Rhode Island, South Dakota, Utah, Vermont, Virginia, Washington, West Virginia, Wisconsin, and Wyoming.

† Variables obtained from infant’s birth certificate include maternal age, race/ethnicity, education level, marital status, WIC participation during pregnancy, health insurance at delivery, number of previous live births, and infant gestational age at birth. Variables obtained from survey include smoked cigarettes during last 3 months of pregnancy, smoked cigarettes postpartum, any intimate partner violence before or during pregnancy, breastfeeding duration, infant vital status at survey completion, self-reported depression before pregnancy, self-reported depression during pregnancy, health care provider asked about depression during prenatal visits, health care provider asked about depression during postpartum visits, and postpartum depressive symptoms.

¶ To provide general guidance on the statistical differences, 95% confidence intervals (CIs) for the prevalence were compared across groups, with an emphasis on identifying differences (i.e., nonoverlap of CIs) between categories within the selected variables. This typically conservative approach might fail to note differences between estimates more often than formal statistical testing. Overlap between CIs does not necessarily mean that there is no statistical difference between estimates.

** The 16 sites include Alaska, Delaware, Illinois, Maine, Massachusetts, Missouri, New Jersey, New Mexico, New York City, Pennsylvania, Utah, Vermont, Washington, West Virginia, Wisconsin, and Wyoming.

†† The 22 sites include Alaska, Colorado, Connecticut, Delaware, Illinois, Louisiana, Maine, Massachusetts, Michigan, Missouri, New Jersey, New Mexico, New York City, Pennsylvania, Rhode Island, Utah, Vermont, Virginia, Washington, West Virginia, Wisconsin, and Wyoming.

§§ https://safehealthcareforeverywoman.org/wp-content/uploads/2017/11/Maternal-Mental-Health-Bundle.pdf;.https://www.jognn.org/article/S0884-2175(17)30001-1/fulltext

¶¶ https://www.hrsa.gov/about/news/press-releases/hrsa-awards-12-million-maternal-child-mental-health-programs#behavioral.

*** National Healthy Start Association. Stress and Depression Training Toolkit. http://nationalhealthystart.org/member/training.

††† https://www.childwelfare.gov/topics/preventing/prevention-programs/homevisit/maternal-mental-health/.

§§§ https://mchb.tvisdata.hrsa.gov/PrioritiesAndMeasures/NationalOutcomeMeasures.
